# Crystal structure of the deuterated hepta­hydrate of potassium phosphate, K_3_PO_4_·7D_2_O

**DOI:** 10.1107/S2056989020000201

**Published:** 2020-01-10

**Authors:** Matthias Weil, Berthold Stöger

**Affiliations:** a Institute for Chemical Technologies and Analytics, Division of Structural Chemistry, Vienna University of Technology, Getreidemarkt 9/164-SC, A-1060 Vienna, Austria; b X-Ray Centre, Getreidemarkt 9, A-1060 Vienna, Austria

**Keywords:** crystal structure, potassium phosphate, hydrogen bonding, absolute structure, hydrate

## Abstract

Deuterated potassium orthophosphate hepta­hydrate, K_3_PO_4_·7D_2_O, crystallizes in the Sohnke space group *P*2_1_, and its absolute structure was determined from 2017 Friedel pairs [Flack parameter 0.004 (16)]. Each of the three crystallographically unique K^+^ cations is surrounded by six water mol­ecules and one oxygen atom from the orthophosphate group, using a threshold for K—O bonds of 3.10 Å. The highly irregular coordination polyhedra are linked by corner- and edge-sharing into a three-dimensional network that is consolidated by an intricate network of O—D⋯O hydrogen bonds of medium strength.

## Abstract 2

An intricate network of medium-strong O—D⋯O hydrogen bonds consolidates the crystal structure of water-rich K_3_PO_4_·7D_2_O.

## Chemical context   

Following projects devoted to studying the formation and crystal chemistry of hydrous arsenate and phosphate phases of monovalent metals, *viz*. NaH_2_AsO_4_ (Ring *et al.*, 2017 ▸), K_2_HAsO_4_(H_2_O)_2.5_ and K_2_HAsO_4_(H_2_O)_6_ (Stöger *et al.*, 2012 ▸), *M*_2_H*X*O_4_·2H_2_O (*M* = Rb, Cs; *X* = P, As; Stöger & Weil, 2014 ▸), and several acidic thallium(I) arsenate phases (Schroffenegger *et al.*, 2019 ▸), we became inter­ested in the system K_3_PO_4_/H_2_O. Although hydrate phases of potassium orthophosphate have been known for a very long time to exist for the 3-hydrate and the 7-hydrate (Gmelin, 1938 ▸), crystal-structure determinations of these two phases or of any other hydrate of K_3_PO_4_ have not been reported so far. Previous investigations on the trihydrate revealed that the crystal structure of K_3_PO_4_·3H_2_O is incommensurately modulated below 300 K (Stöger, 2020 ▸). To better elucidate the role of hydrogen bonding in this structure with the aid of single-crystal neutron diffraction, we started crystal-growth experiments to obtain the deuterium analogue K_3_PO_4_·3D_2_O. The title compound, K_3_PO_4_·7D_2_O, was the unexpected product of such a crystallization attempt at temperatures below the freezing point of pure water, and its crystal structure is reported here.

## Structural commentary   

**Figure 1:**
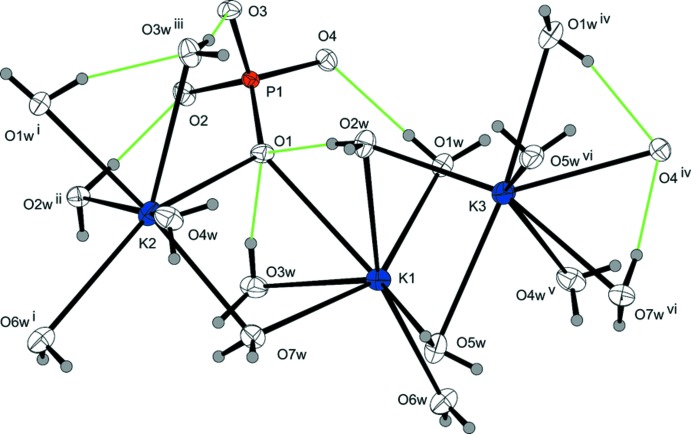
The expanded asymmetric unit of K_3_PO_4_·7D_2_O showing the complete potassium coordination polyhedra. Displacement ellipsoids are displayed at the 74% probability level; O—D⋯O hydrogen bonds are indicated by green lines; symmetry codes refer to Table 1 ▸.

**Figure 2:**
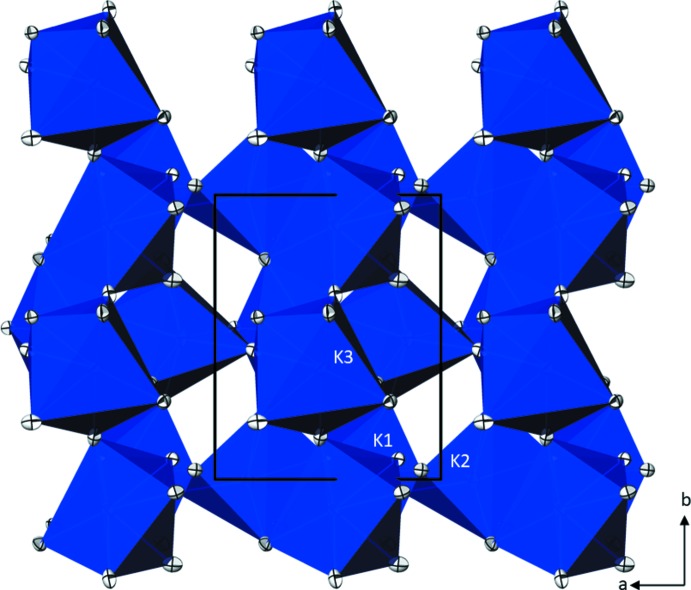
Network of corner- and edge-sharing [KO_7_] polyhedra in the crystal structure of K_3_PO_4_·7D_2_O, viewed along [00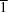]. Displacement ellipsoids are displayed at the 90% probability level. For clarity, D atoms are not shown.

**Figure 3:**
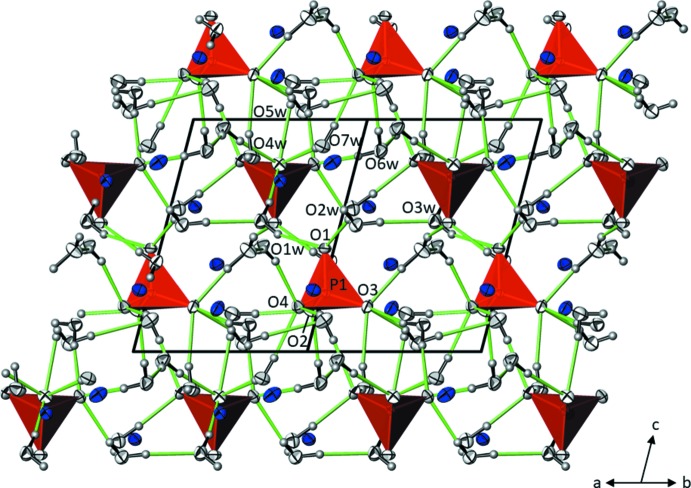
O—D⋯O hydrogen-bonding network (green lines) in the crystal structure of K_3_PO_4_·7D_2_O, viewed along [101]. Displacement ellipsoids are displayed at the 90% probability level.

Taking 3.1 Å as the upper limit of K—O bond lengths in the first coordination sphere, each of the three crystallographically independent potassium cations is surrounded by six water mol­ecules and one oxygen atom of the phosphate group (Fig. 1 ▸). The highly irregular coordination polyhedra show K—O bond lengths ranging between 2.6665 (9) and 3.0151 Å (Table 1 ▸). The overall mean of 2.821 Å for the 21 bonds is in good agreement with the value of 2.861 Å calculated from 469 individual K—O bonds in crystal structures with coordination numbers of 7 for the potassium cation (Gagné & Hawthorne, 2016 ▸). The [K(D_2_O)_6_O] polyhedra share corners and edges to build up a three-dimensional network (Fig. 2 ▸). Each water mol­ecule is a donor group of two slightly bent O—D⋯O hydrogen bonds, but only two of the water mol­ecules (O3*w*, O6*w*) also serve as acceptor groups for one hydrogen bond. All other hydrogen bonds are directed towards the O atoms of the phosphate group, with O1 being twofold, O2 threefold, O3 fourfold and O4 threefold acceptor atoms, respectively (Fig. 3 ▸). Judging from the O⋯O distances [range 2.6931 (12)–2.9025 (13) Å; Table 2 ▸], hydrogen bonds of medium strength are formed in the crystal structure. The PO_4_ tetra­hedron shows almost equal P—O bond lengths typical of a fully deprotonated orthophosphate group (mean 1.546 Å), with marginal angular distortions.

A bond-valence analysis (Brown, 2002 ▸), using the parameters of Brese & O’Keeffe (1991 ▸), reveals bond-valence sums (BVS, in valence units) of K1 = 1.18, K2 = 1.08, K3 = 1.11, and P1 = 4.85, in good agreement with the expected values of +1 and +5, respectively. The four oxygen atoms of the orthophosphate tetra­hedron are considerably underbonded and show BVS values of 1.53 (O1), 1.22 (O2), 1.10 (O3) and 1.38 (O4). O1 with the highest BVS of the four phosphate O atoms has two K^+^ cations as additional bonding partners, O4 with the second highest BVS has one additional K^+^ as bonding partner whereas O2 and O3 with the lowest BVS values are solely bonded to the P atom. The four O atoms compensate for underbonding by means of their role as acceptor atoms in hydrogen bonding (see above).

## Database survey   

In the Inorganic Structure Database (ICSD; Zagorac *et al.*, 2019 ▸), the crystal structures of not less than 14 different phases in the system K_2_O/P_2_O_5_/H_2_O are listed, including partly protonated PO_4_ or other condensed phosphate groups, and/or phases with water mol­ecules. The only other phosphates of an alkali metal, thallium or ammonium with a fully deprotonated orthophosphate group are Na_3_PO_4_(H_2_O)_8_ (Larbot & Durand, 1983 ▸), Na_3_PO_4_(H_2_O)_0.5_ (Averbuch-Pouchot & Durif, 1983 ▸) and (NH_4_)_3_(PO_4_)·3H_2_O (Mootz & Wunderlich, 1970 ▸). As a result of the different size of the Na^+^ cation compared to K^+^, the role of NH_4_^+^ as an active species in hydrogen bonding, and the different amounts of water mol­ecules in these three crystal structures, there is no evident structural relation to K_3_PO_4_·7D_2_O.

## Synthesis and crystallization   

Commercial anhydrous K_3_PO_4_ (Sigma–Aldrich) was dissolved in a small amount of warm D_2_O. Cooling to 255 K afforded rod-like crystals of the title hepta­hydrate that grew over night, with maximum edge lengths in the millimetre range.

## Refinement   

Crystal data, data collection and structure refinement details are summarized in Table 3 ▸. Positions of the D atoms were located in a difference-Fourier map and were refined freely under consideration of scattering factors for hydrogen atoms.

## Supplementary Material

Crystal structure: contains datablock(s) global, I. DOI: 10.1107/S2056989020000201/hb7876sup1.cif

Structure factors: contains datablock(s) I. DOI: 10.1107/S2056989020000201/hb7876Isup2.hkl

CCDC reference: 1976170

Additional supporting information:  crystallographic information; 3D view; checkCIF report

## Figures and Tables

**Table 1:** Selected bond lengths (Å)

K1—O5*w*	2.7153 (10)	K2—O6*w* ^i^	3.0151 (10)
K1—O1*w*	2.7183 (11)	K3—O2*w*	2.6665 (9)
K1—O7*w*	2.7381 (10)	K3—O4^iv^	2.7867 (9)
K1—O6*w*	2.7532 (9)	K3—O4*w* ^v^	2.7983 (10)
K1—O3*w*	2.8479 (9)	K3—O5*w* ^vi^	2.8344 (10)
K1—O2*w*	2.8486 (9)	K3—O1*w* ^iv^	2.8394 (10)
K1—O1	2.9757 (9)	K3—O7*w* ^vi^	2.9094 (9)
K2—O1	2.7317 (10)	K3—O5*w*	2.9246 (10)
K2—O4*w*	2.7391 (10)	P1—O1	1.5414 (8)
K2—O7*w*	2.7659 (9)	P1—O2	1.5440 (8)
K2—O1*w* ^i^	2.7836 (9)	P1—O4	1.5472 (10)
K2—O2*w* ^ii^	2.8269 (9)	P1—O3	1.5523 (8)
K2—O3*w* ^iii^	3.0144 (9)		

Symmetry codes: (i) 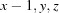; (ii) 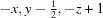; (iii) 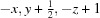; (iv) 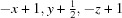; (v) 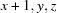; (vi) 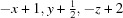.

**Table 2:** Hydrogen-bond geometry (Å, °)

*D*—H⋯*A*	*D*—H	H⋯*A*	*D*⋯*A*	*D*—H⋯*A*
O1*w*—D11⋯O3*w* ^iv^	0.86 (2)	1.91 (2)	2.7255 (13)	158 (2)
O1*w*—D12⋯O4	0.78 (2)	1.98 (2)	2.7391 (11)	164 (2)
O2*w*—D21⋯O2^iii^	0.87 (2)	1.85 (2)	2.7149 (13)	177 (2)
O2*w*—D22⋯O1	0.73 (2)	1.99 (2)	2.7029 (11)	167 (3)
O3*w*—D31⋯O1	0.80 (2)	1.97 (2)	2.7242 (11)	159 (2)
O3*w*—D32⋯O3^ii^	0.98 (3)	1.77 (3)	2.7395 (14)	171 (2)
O4*w*—D41⋯O3^vii^	0.80 (2)	2.10 (2)	2.8870 (14)	169 (2)
O4*w*—D42⋯O2^iii^	0.79 (2)	1.97 (2)	2.7679 (13)	177 (2)
O5*w*—D51⋯O6*w* ^vi^	0.80 (2)	2.11 (2)	2.9025 (13)	168 (2)
O5*w*—D52⋯O3^viii^	0.80 (2)	1.92 (2)	2.6944 (12)	166 (2)
O6*w*—D61⋯O2^viii^	0.75 (2)	1.96 (2)	2.7087 (12)	176 (2)
O6*w*—D62⋯O4^ix^	0.85 (2)	1.86 (2)	2.6931 (12)	165 (2)
O7*w*—D71⋯O3^ii^	0.78 (2)	2.02 (2)	2.7498 (12)	155 (2)
O7*w*—D72⋯O4^vii^	0.82 (2)	1.97 (2)	2.7859 (13)	171 (2)

Symmetry codes: (ii) 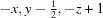; (iii) 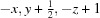; (iv) 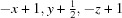; (vi) 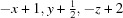; (vii) 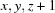; (viii) 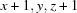; (ix) 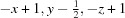.

**Table 3:** Experimental details

Crystal data	
Chemical formula	K_3_PO_4_·7D_2_O
*M*_r_	352.5
Crystal system, space group	Monoclinic, *P*2_1_
Temperature (K)	100
*a*, *b*, *c* (Å)	7.8325 (7), 9.3406 (8), 8.4471 (7)
β (°)	108.727 (2)
*V* (Å^3^)	585.28 (9)
*Z*	2
Radiation type	Mo *K*α
μ (mm^−1^)	1.34
Crystal size (mm)	0.46 × 0.09 × 0.01

Data collection	
Diffractometer	Bruker Kappa APEXII CCD
Absorption correction	Multi-scan (*SADABS*; Bruker, 2016 ▸)
*T*_min_, *T*_max_	0.54, 0.99
No. of measured, independent and observed [*I* > 3σ(*I*)] reflections	9464, 4273, 4127
*R*_int_	0.021
(sin θ/λ)_max_ (Å^−1^)	0.759

Refinement	
*R*[*F* ^2^ > 2σ(*F* ^2^)], *wR*(*F* ^2^), *S*	0.016, 0.020, 1.02
No. of reflections	4273
No. of parameters	193
Δρ_max_, Δρ_min_ (e Å^−3^)	0.16, −0.13
Absolute structure	2017 Friedel pairs used in the refinement (Flack, 1983 ▸)
Absolute structure parameter	0.004 (16)

Computer programs: *APEX2* and *SAINT-Plus* (Bruker, 2016 ▸), *SHELXT* (Sheldrick, 2015 ▸), *JANA2006* (Petříček *et al.*, 2014 ▸), *ATOMS* (Dowty, 2006 ▸) and *publCIF* (Westrip, 2010 ▸).

## supplementary crystallographic information

### Crystal data

**Table:**

K_3_PO_4_·7D_2_O	*F*(000) = 348
*M**_r_* = 352.5	*D*_x_ = 2.000 Mg m^−^^3^
Monoclinic, *P*2_1_	Mo *K*α radiation, λ = 0.71073 Å
Hall symbol: P 2yb	Cell parameters from 7382 reflections
*a* = 7.8325 (7) Å	θ = 2.6–32.6°
*b* = 9.3406 (8) Å	µ = 1.34 mm^−^^1^
*c* = 8.4471 (7) Å	*T* = 100 K
β = 108.727 (2)°	Rod, colourless
*V* = 585.28 (9) Å^3^	0.46 × 0.09 × 0.01 mm
*Z* = 2	

### Data collection

**Table:**

Bruker Kappa APEXII CCD diffractometer	4273 independent reflections
Radiation source: X-ray tube	4127 reflections with *I* > 3σ(*I*)
Graphite monochromator	*R*_int_ = 0.021
ω– and φ–scans	θ_max_ = 32.6°, θ_min_ = 2.6°
Absorption correction: multi-scan (*SADABS*; Bruker, 2016)	*h* = −10→11
*T*_min_ = 0.54, *T*_max_ = 0.99	*k* = −14→14
9464 measured reflections	*l* = −12→10

### Refinement

**Table:**

Refinement on *F*	1 constraint
*R*[*F*^2^ > 2σ(*F*^2^)] = 0.016	Weighting scheme based on measured s.u.'s *w* = 1/(σ^2^(*F*) + 0.0001*F*^2^)
*wR*(*F*^2^) = 0.020	(Δ/σ)_max_ = 0.019
*S* = 1.02	Δρ_max_ = 0.16 e Å^−^^3^
4273 reflections	Δρ_min_ = −0.13 e Å^−^^3^
193 parameters	Absolute structure: 2017 Friedel pairs used in the refinement (Flack, 1983)
0 restraints	Absolute structure parameter: 0.004 (16)

### Fractional atomic coordinates and isotropic or equivalent isotropic displacement parameters (Å^2^)

**Table:**

	*x*	*y*	*z*	*U*_iso_*/*U*_eq_	
K1	0.42550 (3)	0.01804 (2)	0.73444 (3)	0.00950 (6)	
K2	−0.14458 (3)	−0.00537 (2)	0.62341 (3)	0.00990 (6)	
K3	0.52865 (3)	0.39152 (2)	0.81480 (3)	0.01224 (6)	
P1	0.02505 (3)	0.04285 (3)	0.25261 (3)	0.00571 (7)	
O1	0.08740 (10)	0.03291 (8)	0.44490 (9)	0.00823 (19)	
O2	−0.06671 (10)	−0.09902 (8)	0.17723 (10)	0.0101 (2)	
O3	−0.10938 (10)	0.16905 (8)	0.19358 (10)	0.0092 (2)	
O4	0.18924 (10)	0.07033 (8)	0.19270 (10)	0.0086 (2)	
O1w	0.50577 (11)	0.08478 (8)	0.45314 (11)	0.0119 (2)	
O2w	0.22528 (11)	0.27301 (8)	0.61723 (11)	0.0106 (2)	
O3w	0.23569 (11)	−0.22553 (8)	0.56243 (11)	0.0116 (2)	
O4w	−0.18873 (12)	0.20109 (9)	0.83725 (11)	0.0129 (2)	
O5w	0.53272 (11)	0.15028 (9)	1.03669 (11)	0.0126 (2)	
O6w	0.71314 (11)	−0.16355 (8)	0.86601 (11)	0.0119 (2)	
O7w	0.17243 (11)	−0.04581 (9)	0.88427 (10)	0.0103 (2)	
D11	0.564 (2)	0.161 (2)	0.442 (2)	0.027 (5)*	
D12	0.409 (3)	0.091 (2)	0.390 (3)	0.033 (5)*	
D21	0.172 (3)	0.315 (2)	0.680 (2)	0.034 (5)*	
D22	0.173 (3)	0.213 (2)	0.570 (3)	0.037 (6)*	
D31	0.182 (2)	−0.162 (2)	0.506 (3)	0.031 (5)*	
D32	0.179 (3)	−0.261 (3)	0.642 (3)	0.052 (7)*	
D41	−0.153 (2)	0.1885 (19)	0.936 (3)	0.022 (4)*	
D42	−0.113 (3)	0.258 (3)	0.837 (3)	0.049 (7)*	
D51	0.477 (2)	0.2098 (19)	1.068 (2)	0.021 (4)*	
D52	0.634 (2)	0.1654 (19)	1.093 (2)	0.023 (4)*	
D61	0.776 (3)	−0.143 (2)	0.950 (3)	0.031 (5)*	
D62	0.759 (2)	−0.240 (2)	0.842 (2)	0.023 (4)*	
D71	0.132 (2)	−0.123 (2)	0.877 (2)	0.023 (4)*	
D72	0.166 (2)	−0.0150 (19)	0.973 (2)	0.019 (4)*	

### Atomic displacement parameters (Å^2^)

**Table:**

	*U*^11^	*U*^22^	*U*^33^	*U*^12^	*U*^13^	*U*^23^
K1	0.01055 (8)	0.00951 (9)	0.00895 (9)	0.00141 (6)	0.00384 (7)	0.00041 (7)
K2	0.00815 (8)	0.01323 (9)	0.00836 (9)	0.00028 (7)	0.00272 (7)	−0.00109 (7)
K3	0.01205 (9)	0.00943 (9)	0.01200 (11)	−0.00261 (7)	−0.00067 (8)	0.00104 (7)
P1	0.00639 (10)	0.00552 (10)	0.00530 (11)	−0.00037 (8)	0.00201 (8)	0.00003 (8)
O1	0.0102 (3)	0.0084 (3)	0.0056 (3)	0.0002 (2)	0.0020 (2)	−0.0001 (2)
O2	0.0131 (3)	0.0080 (3)	0.0086 (3)	−0.0040 (3)	0.0027 (3)	−0.0020 (3)
O3	0.0086 (3)	0.0090 (3)	0.0099 (4)	0.0029 (2)	0.0028 (3)	0.0022 (3)
O4	0.0089 (3)	0.0088 (3)	0.0092 (4)	−0.0002 (2)	0.0046 (3)	0.0004 (2)
O1w	0.0078 (3)	0.0150 (4)	0.0114 (4)	−0.0011 (3)	0.0010 (3)	0.0026 (3)
O2w	0.0091 (3)	0.0103 (3)	0.0120 (4)	−0.0011 (3)	0.0026 (3)	−0.0033 (3)
O3w	0.0127 (3)	0.0109 (3)	0.0123 (4)	0.0032 (3)	0.0056 (3)	0.0032 (3)
O4w	0.0182 (4)	0.0112 (3)	0.0102 (4)	−0.0007 (3)	0.0059 (3)	0.0003 (3)
O5w	0.0084 (3)	0.0168 (4)	0.0122 (4)	−0.0016 (3)	0.0030 (3)	−0.0033 (3)
O6w	0.0111 (3)	0.0099 (3)	0.0122 (4)	0.0020 (3)	0.0003 (3)	−0.0024 (3)
O7w	0.0126 (3)	0.0098 (3)	0.0091 (4)	−0.0010 (3)	0.0041 (3)	−0.0008 (3)

### Geometric parameters (Å, º)

**Table:**

K1—O5w	2.7153 (10)	K3—O5w	2.9246 (10)
K1—O1w	2.7183 (11)	P1—O1	1.5414 (8)
K1—O7w	2.7381 (10)	P1—O2	1.5440 (8)
K1—O6w	2.7532 (9)	P1—O4	1.5472 (10)
K1—O3w	2.8479 (9)	P1—O3	1.5523 (8)
K1—O2w	2.8486 (9)	O1w—D11	0.86 (2)
K1—O1	2.9757 (9)	O1w—D12	0.778 (18)
K2—O1	2.7317 (10)	O2w—D21	0.87 (2)
K2—O4w	2.7391 (10)	O2w—D22	0.73 (2)
K2—O7w	2.7659 (9)	O3w—D31	0.796 (19)
K2—O1w^i^	2.7836 (9)	O3w—D32	0.98 (3)
K2—O2w^ii^	2.8269 (9)	O4w—D41	0.80 (2)
K2—O3w^iii^	3.0144 (9)	O4w—D42	0.79 (2)
K2—O6w^i^	3.0151 (10)	O5w—D51	0.80 (2)
K3—O2w	2.6665 (9)	O5w—D52	0.795 (17)
K3—O4^iv^	2.7867 (9)	O6w—D61	0.748 (19)
K3—O4w^v^	2.7983 (10)	O6w—D62	0.85 (2)
K3—O5w^vi^	2.8344 (10)	O7w—D71	0.783 (19)
K3—O1w^iv^	2.8394 (10)	O7w—D72	0.823 (19)
K3—O7w^vi^	2.9094 (9)		
			
O1—K1—O1w	70.45 (2)	O1w^iv^—K3—O5w^vi^	79.89 (3)
O1—K1—O2w	55.25 (2)	O1w^iv^—K3—O7w^vi^	114.35 (2)
O1—K1—O3w	55.73 (2)	O2w—K3—O4w^v^	107.76 (3)
O1—K1—O5w	132.58 (3)	O2w—K3—O5w	84.57 (2)
O1—K1—O6w	139.24 (2)	O2w—K3—O5w^vi^	112.79 (3)
O1—K1—O7w	78.74 (2)	O2w—K3—O7w^vi^	159.36 (3)
O1w—K1—O2w	76.14 (3)	O4w^v^—K3—O5w	67.74 (3)
O1w—K1—O3w	88.09 (3)	O4w^v^—K3—O5w^vi^	139.02 (2)
O1w—K1—O5w	128.98 (3)	O4w^v^—K3—O7w^vi^	70.82 (3)
O1w—K1—O6w	96.04 (3)	O5w—K3—O5w^vi^	109.99 (3)
O1w—K1—O7w	149.19 (2)	O5w—K3—O7w^vi^	75.81 (2)
O2w—K1—O3w	110.59 (2)	O5w^vi^—K3—O7w^vi^	69.21 (2)
O2w—K1—O5w	85.18 (3)	O1—P1—O2	109.32 (4)
O2w—K1—O6w	160.60 (2)	O1—P1—O3	109.69 (5)
O2w—K1—O7w	86.75 (3)	O1—P1—O4	109.83 (4)
O3w—K1—O5w	142.78 (3)	O2—P1—O3	109.96 (4)
O3w—K1—O6w	86.52 (2)	O2—P1—O4	109.46 (5)
O3w—K1—O7w	74.04 (3)	O3—P1—O4	108.56 (4)
O5w—K1—O6w	86.26 (3)	K2—O1—P1	123.21 (4)
O5w—K1—O7w	73.48 (3)	K3^vii^—O4—P1	131.03 (4)
O6w—K1—O7w	107.41 (3)	K1—O1w—K2^v^	86.80 (2)
O1—K2—O1w^i^	113.18 (3)	K1—O1w—K3^vii^	124.16 (3)
O1—K2—O2w^ii^	74.56 (3)	K2^v^—O1w—K3^vii^	92.60 (3)
O1—K2—O3w^iii^	71.77 (3)	K1—O2w—K2^iii^	146.43 (4)
O1—K2—O4w	121.27 (3)	K1—O2w—K3	81.32 (2)
O1—K2—O6w^i^	153.61 (2)	K2^iii^—O2w—K3	95.43 (3)
O1—K2—O7w	82.62 (3)	D21—O2w—D22	113 (3)
O1w^i^—K2—O2w^ii^	83.93 (2)	K1—O3w—D31	78.3 (13)
O1w^i^—K2—O3w^iii^	55.91 (2)	K1—O3w—D32	101.6 (14)
O1w^i^—K2—O4w	79.41 (3)	D31—O3w—D32	114 (2)
O1w^i^—K2—O6w^i^	88.99 (3)	K2—O4w—K3^i^	131.87 (3)
O1w^i^—K2—O7w	159.32 (3)	K2—O4w—D41	120.6 (14)
O2w^ii^—K2—O3w^iii^	107.43 (3)	K2—O4w—D42	102.2 (19)
O2w^ii^—K2—O4w	160.62 (3)	K3^i^—O4w—D41	99.7 (15)
O2w^ii^—K2—O6w^i^	94.81 (3)	K3^i^—O4w—D42	98.8 (17)
O2w^ii^—K2—O7w	114.15 (3)	D41—O4w—D42	95 (2)
O3w^iii^—K2—O4w	70.97 (3)	K1—O5w—K3^viii^	89.03 (2)
O3w^iii^—K2—O6w^i^	134.55 (3)	K1—O5w—D51	126.3 (11)
O3w^iii^—K2—O7w	122.36 (2)	K1—O5w—D52	126.4 (16)
O4w—K2—O6w^i^	75.19 (3)	K3^viii^—O5w—D51	104.9 (14)
O4w—K2—O7w	80.90 (3)	K3^viii^—O5w—D52	99.4 (13)
O6w^i^—K2—O7w	79.91 (3)	D51—O5w—D52	102.5 (18)
O4^iv^—K3—O1w^iv^	58.26 (2)	K1—O6w—D61	115.4 (16)
O4^iv^—K3—O2w	142.03 (3)	K1—O6w—D62	140.5 (11)
O4^iv^—K3—O4w^v^	76.51 (3)	D61—O6w—D62	104 (2)
O4^iv^—K3—O5w	129.11 (2)	K1—O7w—K2	101.53 (3)
O4^iv^—K3—O5w^vi^	75.37 (3)	K1—O7w—D71	120.0 (16)
O4^iv^—K3—O7w^vi^	58.52 (2)	K1—O7w—D72	127.9 (12)
O1w^iv^—K3—O2w	85.85 (3)	K2—O7w—D71	80.0 (11)
O1w^iv^—K3—O4w^v^	109.19 (3)	K2—O7w—D72	112.2 (11)
O1w^iv^—K3—O5w	168.35 (2)	D71—O7w—D72	105 (2)

Symmetry codes: (i) *x*−1, *y*, *z*; (ii) −*x*, *y*−1/2, −*z*+1; (iii) −*x*, *y*+1/2, −*z*+1; (iv) −*x*+1, *y*+1/2, −*z*+1; (v) *x*+1, *y*, *z*; (vi) −*x*+1, *y*+1/2, −*z*+2; (vii) −*x*+1, *y*−1/2, −*z*+1; (viii) −*x*+1, *y*−1/2, −*z*+2.

### Hydrogen-bond geometry (Å, º)

**Table:**

*D*—H···*A*	*D*—H	H···*A*	*D*···*A*	*D*—H···*A*
O1*w*—D11···O3*w*^iv^	0.86 (2)	1.91 (2)	2.7255 (13)	158 (2)
O1*w*—D12···O4	0.78 (2)	1.98 (2)	2.7391 (11)	164 (2)
O2*w*—D21···O2^iii^	0.87 (2)	1.85 (2)	2.7149 (13)	177 (2)
O2*w*—D22···O1	0.73 (2)	1.99 (2)	2.7029 (11)	167 (3)
O3*w*—D31···O1	0.80 (2)	1.97 (2)	2.7242 (11)	159 (2)
O3*w*—D32···O3^ii^	0.98 (3)	1.77 (3)	2.7395 (14)	171 (2)
O4*w*—D41···O3^ix^	0.80 (2)	2.10 (2)	2.8870 (14)	169 (2)
O4*w*—D42···O2^iii^	0.79 (2)	1.97 (2)	2.7679 (13)	177 (2)
O5*w*—D51···O6*w*^vi^	0.80 (2)	2.11 (2)	2.9025 (13)	168 (2)
O5*w*—D52···O3^x^	0.80 (2)	1.92 (2)	2.6944 (12)	166 (2)
O6*w*—D61···O2^x^	0.75 (2)	1.96 (2)	2.7087 (12)	176 (2)
O6*w*—D62···O4^vii^	0.85 (2)	1.86 (2)	2.6931 (12)	165 (2)
O7*w*—D71···O3^ii^	0.78 (2)	2.02 (2)	2.7498 (12)	155 (2)
O7*w*—D72···O4^ix^	0.82 (2)	1.97 (2)	2.7859 (13)	171 (2)

Symmetry codes: (ii) −*x*, *y*−1/2, −*z*+1; (iii) −*x*, *y*+1/2, −*z*+1; (iv) −*x*+1, *y*+1/2, −*z*+1; (vi) −*x*+1, *y*+1/2, −*z*+2; (vii) −*x*+1, *y*−1/2, −*z*+1; (ix) *x*, *y*, *z*+1; (x) *x*+1, *y*, *z*+1.
